# Investigation of Self-Assembled Flexible Zein Nanoparticles and Their Sensitivity to Complex Environments

**DOI:** 10.3390/foods14050859

**Published:** 2025-03-03

**Authors:** Shirong Dong, Guangqing Mu

**Affiliations:** 1 School of Food Science and Technology, Dalian Polytechnic University, Dalian 116034, China; dongshirong118@126.com; 2 School of Food Engineering, Harbin University, Harbin 150086, China

**Keywords:** zein, flexibility, acidic modification, complex environment, sensitivity, nanoparticle

## Abstract

Zein was made flexible through acid-driven deamidation. This increased flexibility was confirmed by the higher release of water-soluble peptides during trypsin hydrolysis. Self-assembled flexible zein nanoparticles (FZNPs) were prepared using the anti-solvent precipitation method. To test the sensitivity of FZNPs to complex environment, ionic solutions (CaCl_2_ and NaCl) at various concentrations were prepared. The morphology and particle size of FZNPs differed significantly from those of control zein nanoparticles (NZNPs). As the ionic concentration increased from 0 to 15 mmol/L, FZNPs showed higher electrical conductivity and adsorption capacity than NZNPs. This suggests that FZNPs are highly sensitive to complex environment. X-Ray Photoelectron Spectrum (XPS) results revealed that both FZNPs and NZNPs bound more Na^+^ than Ca^2+^. The enhanced sensitivity of FZNPs to complex environments may be due to their greater tendency for structural changes. These conformational changes are likely caused by the altered amino acids in flexible zein, which result from deamidation. This study offers a practical approach to designing novel nanoparticles as functional materials for delivering bioactive compounds.

## 1. Introduction

In recent years, plant-based proteins have garnered significant attention due to numerous advantageous properties, including low cost, abundance, amphipathic nature, biodegradability, biocompatibility, and nontoxicity [1,2,3]. Zein, a predominant storage protein found in corn, stands out for its unique characteristic. Comprising over 50% hydrophobic amino acid residues, zein exhibits insolubility in pure water but demonstrates solubility in aqueous ethanol solutions ranging from 60 to 90% [4,5]. The distinctive amino acid sequence and molecular structure endow zein with a pronounced amphipathic character, facilitating the formation of self-assembled zein aggregates [1]. The anti-solvent precipitation method, also known as the liquid-liquid dispersion method, has been extensively employed to fabricate self-assembled zein nanoparticles [5,6,7,8]. These nanoparticles are recognized as valuable biomaterials, primarily owing to their exceptional safety profile [9]. Nevertheless, the stability of zein nanoparticles is susceptible to various environmental stresses, such as pH near the isoelectric point (pI ≈ 6.2), high ionic strength and heat treatment [4,7,10].

To enhance the environmental adaptability of zein nanoparticles, an effective strategy involves modifying their microstructure and particle size through complexation with polyphenols [2], polysaccharides [4], and proteins [7]. Additionally, chemical modifications, such as acidic or alkali treatment, and enzymatic modifications have been employed to promote deamidation reactions, thereby improving the environmental adaptability of zein nanoparticles. Deamidation primarily removes amide groups from glutamine and asparagine residues in gluten proteins, converting them into acidic residues. This process, particularly under acidic and high-temperature conditions, increases electrostatic repulsion, leading to changes in protein conformation. The resulting increase in negatively charged polar groups enhances the protein’s amphiphilicity [11]. While various acids can induce protein deamidation, the functional properties of the deamidated proteins vary depending on the acid used. For instance, differences have been observed between the performance of HCl-deamidated gliadin and citric acid-deamidated gliadin in emulsions [11,12]. In our previous studies, alkaline modification yielded flexible zein, and the resulting flexible zein nanoparticles (FZNPs) exhibited excellent environmental adaptability [8,13]. Protein flexibility can be defined as the relative movement of various domains within the protein or the reorientational relaxation rates of amino acid residues in its polypeptide chains [14]. Zein possesses a unique molecular structure, characterized by a prism model consisting of 9–10 helical segments aligned in an antiparallel fashion, connected by glutamine-rich bridges [15]. Deamidation disrupts these glutamine-rich bridges by converting amide groups (glutamine or asparagine) to acid groups (glutamic acid and aspartic acid), thereby reducing interaction among the aligned antiparallel and increasing the flexibility of zein molecules [8,13]. These conversions also enhance the surface hydrophobicity of zein, improving interactions between the protein molecules, and facilitating the formation of zein nanoparticles [1,16]. A higher degree of deamidation induces structural changes in zein, such as reduction in α-helix content [1,17]. However, excessive deamidation can hinder the formation of zein nanoparticles. An optimal degree of deamidation is beneficial for zein nanoparticles formation, promoting the expansion and redistribution of protein emulsions at oil-water interface [13]. The flexibility of zein, reflected in its ability to adapt its structure to environmental changes, is crucial for its functionality. Greater flexibility enhances the sensitivity of zein to environmental variations [14,18]. Despite these advantages, alkaline deamidation has drawbacks, including the destruction of cysteine, leading to the formation of lysine and alanine residues, and the reduction of L-enantiomers and digestibility of essential amino acids. Toxicological studies have shown that these changes can cause kidney damage in mice [19,20]. Consequently, acidic conditions are often preferred for protein deamidation to mitigate these adverse effects.

The impact of acid-driven deamidation on the flexibility enhancement of zein in specific solvents, along with how this flexibility influences its environment adaptability, remains insufficiently explored. Ionic strength, particularly calcium ions, is known to significantly influence the conformational and structural properties of proteins. Notably, CaCl_2_ has been demonstrated to modify the self-assembly capabilities of zein molecules [2]. Given that NaCl is ubiquitous in food processing and present in nearly all food products, a complex environment was simulated using varying concentrations of NaCl and CaCl_2_ solutions.

In this study, flexible zein molecules were synthesized through an acid-driven deamidation reaction, with the degree of flexibility quantified by the enhanced release of water-soluble peptides during trypsin hydrolysis. Hydrochloric acid (HCl) was selected as the deamidating agent. Nanoparticles were subsequently fabricated from these flexible zein molecules using the anti-solvent precipitation method. The flexibility of the acidic-modified zein was assessed and corroborated through an emulsion property analysis. Additionally, the morphology and particle size of the resulting FZNPs were characterized. To evaluate the stability of FZNPs in environments containing monovalent and divalent ions, solutions with varying concentrations of CaCl_2_ and NaCl were prepared. Finally, the structural and amino acid compositional differences between the flexible zein and control zein were compared. This research offers valuable insights into the formation of FZNPs and their responsiveness to complex environments. Moreover, this research holds significant potential for guiding the design of advanced nanoparticles tailored for the delivery of bioactive compounds, as well as for their integration into complex food systems, including but not limited to emulsion-based systems.

## 2. Materials and Methods

### 2.1. Materials

Zein (Z3625), Nile red, Nile blue and trypsin were obtained from Sigma Aldrich (St. Louis, MO, USA). Throughout the experiments, deionized water was utilized. Additional reagents and chemicals, including ethanol, HCl, soy oil, and sulfuric acid, were procured from a local supermarket and were of analytical grade.

### 2.2. Sample Preparation

First, 2.5 g of zein was dissolved into 100 mL of 80% (*v*/*v*) aqueous ethanol solutions to prepare a stock solution (2.5%, *w*/*v*). The stock solution was continuously stirred magnetically at 8000 r/min for 1 h to ensure complete dispersion of zein. The solution was then divided equally into two parts. One part was designated as the control zein, while the other part was adjusted to pH 2.0 using hydrochloric acid (HCl) and heated at 70 °C for 10 h. The resulting acidic-driven deamidated zein was referred to as flexible zein.

**Figure 1:**
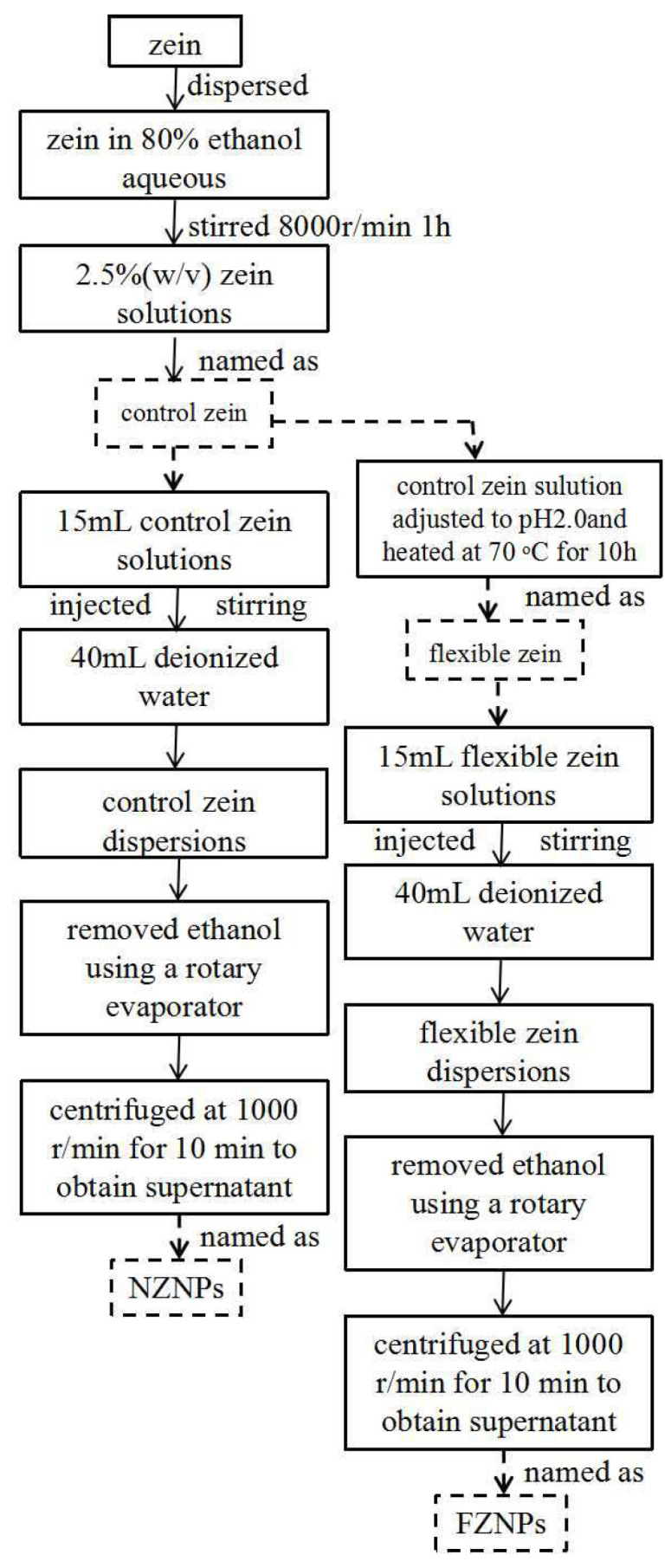
The schematic diagram of sample preparation.

Nanoparticles were prepared using anti-solvent precipitation method [5]. Briefly, approximately 15 mL of the control and flexible zein stock solutions were slowly injected (using a syringe) into 40 mL of deionized water over 2 min under continuous magnetic stirring at 4000 rpm for 30 min to form dispersions. Subsequently, the residual ethanol in the particle dispersions was removed using a rotary evaporator (N1001, Shanghai Ailang Instrument Co., Ltd., Shanghai, China) at 45 °C. The resulting zein nanoparticle dispersions were then centrifuged at 1000 r/min for 10 min to obtain the supernatant. Finally, the nanoparticle dispersions were freeze-dried for 48 h using a freeze dryer (FDU-2110, Tokyo Physical and Chemical Equipment Co., Ltd., Tokyo, Japan). The nanoparticles derived from the control zein and flexible zein were designated as NZNPs and FZNPs, respectively. A schematic diagram of the sample preparation process was shown in Figure 1.

### 2.3. Determination of Flexibility

The flexibility of flexible zein was assessed using the method described by Kato et al. [21]. Briefly, 1 mg/mL solutions of flexible zein were prepared in deionized water. Enzyme solutions (1 mg/mL) were prepared in Tris-HCl buffer (0.05 mol/L, pH 8.0). The enzymatic modification of flexible zein was carried out at a protein-to-enzyme ratio 16:1 (*v*/*v*) at 38 °C for 5 min. The reaction was terminated by adding 4 mL of 5.0% aqueous trichloroacetic acid (TCA). The samples were then centrifuged at 4000 r/min for 30 min, and the amount of hydrolyzed protein in the supernatant was quantified using the BCA method. The degree of protein hydrolysis was used as an indicator of protein flexibility.

### 2.4. Deamidation Degree (DD) Measurement

The DD of FZNPs was determined by the method described by Weatherburn [22]. DD was calculated as the ratio of released ammonia to the total ammonia content. Total ammonia was measured by treating the native zein solution with 3 mol/L H_2_SO_4_.

### 2.5. Confocal Laser Scanning Microscopy (CLSM) Determination

The CLSM observation of NZNPs and FZNPs were performed as previously described [13].

### 2.6. Scanning Electron Microscope (SEM) Experiment

The morphologies of NZNPs and FZNPs were observed using a SEM (SU8010, Hitachi, Tokyo, Japan). The nanoparticles were mounted on conductive carbon tape and sputter-coated with a gold layer to prevent charging under the electron beam.

### 2.7. Particle Size and Zeta Potential Measurement

The particle sizes and zeta potential of NZNPs and FZNPs were measured using a dynamic light scattering (DLS) instrument (Zetasizer Nano ZS, Malvern Instruments, Worcestershire, UK) at 25 °C. The nanoparticle concentrations were adjusted to 0.6% (*w*/*v*) using deionized water. All measurements were performed in triplicate.

### 2.8. Electrical Conductivity and Adsorption Quantity Measurement

To evaluate the sensitivity of FZNPs to complex environment, solutions of NaCl and CaCl_2_ were prepared at concentrations of 0, 2, 4, 6, 8, 10, 12, 15 mmol/L. NZNPs and FZNPs were added to these solutions to achieve a final protein concentration of 10 mg/mL. The dispersions were then centrifuged at 10,000 r/min for 10 min and the supernatants were collected for further analysis.

The electrical conductivity of the supernatants was measured at room temperature using a conductivity meter (Luocheng analytical, Shanghai, China). These supernatants were diluted 100-fold with deionized water, and the calcium content was determined using atomic absorption spectrophotometry (Shanghai spectrum instrument Co., Ltd., Shanghai, China). The amounts of adsorbed calcium and sodium were calculated as the difference between the total calcium content and the calcium content in the supernatants.

### 2.9. X-Ray Photoelectron Spectrum (XPS) Measurement

The surface levels of Ca^2+^ and Na^+^ on FZNPs and NZNPs were analyzed using XPS. The NZNPs were treated with 6 mmol/L and 12 mmol/L CaCl_2_ and NaCl solutions. The peak area for each element (Ca^2+^ or Na^+^) was obtained using Origin8.0 software, which reflects the quantity of the element associated with the nanoparticles. The relative amounts of bound elements were compared based on their peak area [23].

### 2.10. X-Ray Diffraction (XRD)

The crystal morphologies of NZNPs and FZNPs were analyzed using XRD (ultimaIV, Rigaku Corporation, Tokyo, Japan) with a 2θ scanning range of 5–90° and a scanning speed of 5°/min [24].

### 2.11. Circular Dichroism (CD) Analysis

CD spectra (190–260 nm) of NZNPs and FZNPs were recorded using a J-815 spectropolarimeter (Jasco Corporation, Tokyo, Japan) [25]. The sample concentration was adjusted to 0.01 mg/mL using deionized water. Secondary structure differences were qualitatively assessed by comparing the spectra of the nanoparticles. The CD spectra were expressed as mean residue ellipticity [θ] in degrees cm^−2^ dmol^−1^. The α-helix content in NZNPs and FZNPs was calculated using the equation proposed by Yang et al. [26],

(1) $$P_{\alpha -helix}=\frac{-(\theta_{222}+3000)}{33,000}$$

where *θ*_222_ is the mean residue ellipticity at a wavelength of 222 nm.

### 2.12. Amino Acid Analysis

The amino acid compositions of NZNPs and FZNPs were determined by hydrolyzing the samples with 6 mol/L HCl at 110 °C for 24 h in sealed tubes. The hydrolyzed samples were analyzed using an automatic amino acid analyzer (L-880, Hitachi Co. Ltd., Tokyo, Japan) with phenyl isothiocyanate, following the manufacturer’s recommended procedure.

### 2.13. Statistical Analysis

Data were expressed as mean ± standard deviation (SD). Differences among groups were analyzed using one-way analysis of variance (ANOVA) with Tukey’s test. A *p* value less than 0.05 was considered statistically significant.

## 3. Results

### 3.1. Flexibility of the Flexible Zein

Thermal treatment combined with acidic modification induced a deamidation reaction [27], which significantly enhanced the flexibility of zein molecule [13,17]. Specifically, zein was modified through acidic treatment (HCl) and thermal treatment (70 °C) in an 80% ethanol aqueous solution. The resulting acidic-driven deamidated zein exhibited a flexibility of 4.89% and a deamidation degree of 5.83% (Figure 1).

**Figure 2:**
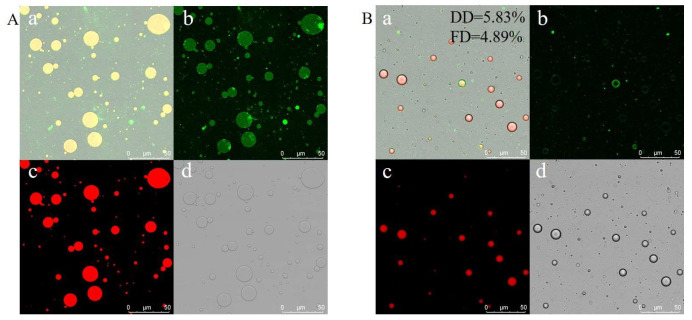
CLSM images of the emulsions stabilized by the control zein (**A**) and flexible zein (**B**). Corn oil was stained with Nile Red (**c**), control zein and flexible zein were stained with Nile Blue (**b**), overlapping drawing (**a**) and bright field (**d**).

Protein flexibility is closely linked to emulsifying properties [28]. As Cabra et al. [16] reported, flexibility is a critical factor influencing a protein’s emulsifying capacity, as it facilitates interactions with oil surface. The adsorption of proteins at interfaces plays a vital role in the formation and stabilization of emulsions. Emulsifying properties are typically evaluated in dynamic system. The ability of a protein to form an emulsion with fine oil droplets depends on its structural rearrangement, which is directly related to molecular flexibility [29,30]. To further validate the flexibility of the acidic-driven deamidated zein, emulsion stability was assessed. These emulsion stability were analyzed using CLSM after 24 h as shown in Figure 2. The CLSM images revealed distinct fluorescence patterns: green fluorescence represented protein areas (Figure 2A(b),B(b)), red fluorescence indicated oil areas (Figure 2A(c),B(c)), and overlapping fluorescence images were obtained by exciting Nile Red and Nile Blue (Figure 2A(a,d),B(a,d)). Visually, the oil droplets stabilized by flexible zein exhibited stronger green fluorescence (Figure 2B), indicating a thicker protein layer surrounding the oil droplets. This suggests that the flexible zein was more effectively absorbed at the interface compared to the control zein. Additionally, emulsions formed from FZNPs displayed smaller particle sizes than those from control zein, further confirming the enhanced flexibility achieved through acid-driven deamidation. These findings collectively demonstrate the modification of zein to improve its flexibility.

### 3.2. SEM Analysis

**Figure 3:**
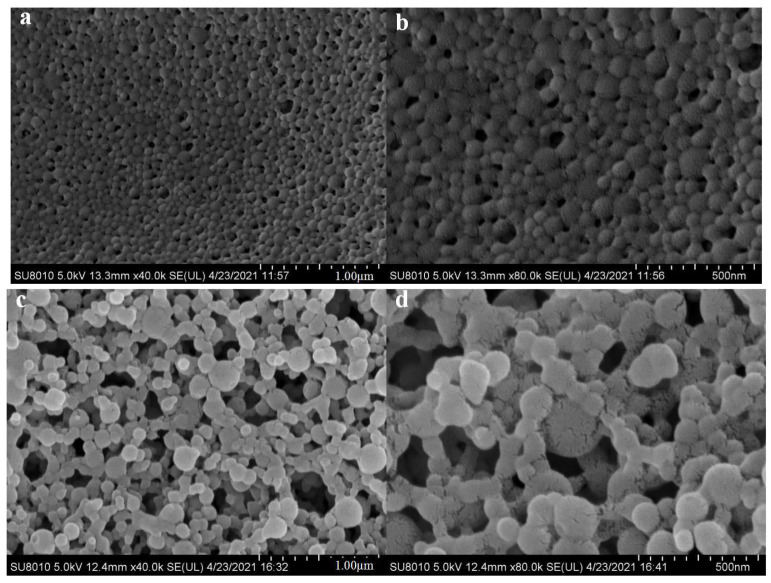
SEM morphologies of the nanoparticles formed from the control and flexible zein; NZNPs magnified at 40.0 k (**a**) and 80.0 k (**b**), FZNPs magnified at 40.0 k (**c**) and 80.0 k (**d**).

The morphology of NZNPs and FZNPs was examined using SEM at magnification of 40.0 k and 80.0 k, as depicted in Figure 3. The NZNPs exhibited a uniform and regular distribution (Figure 3a), maintaining a compact structure even at higher magnification (Figure 3b). In contrast, the FZNPs displayed a less regular distribution (Figure 3c), with some particles appearing fragmented (Figure 3d). Song et al. [31] noted that the addition of glutamic acid to zein altered the size, shape, and distribution of zein particles by promoting intermolecular aggregation. In this study, flexible zein was prepared under acidic conditions without subsequent neutralization to minimize the introduction of additional ions. The pH of the environment played a critical role in influencing the interaction among flexible zein molecules. Yu et al. [1] demonstrated that acidic deamidation converts glutamine to glutamic acid, increasing the surface hydrophobicity of zein, and enhancing the intermolecular interactions. This process, however, reduces the stability of zein particles, leading to their aggregation into microspheres. Similar results had also been reported by Cabra et al. [16]. Additionally, the higher relative hydration of zein under acidic conditions contributes to the formation of larger, more diffuse structures observed in electron microscopy. The distinct morphological differences between NZNPs and FZNPs can be attributed to changes in molecular structure, electrostatic properties and acidic conditions resulting from the modification process.

### 3.3. Particle Size and Zeta Potential Analysis

**Figure 4:**
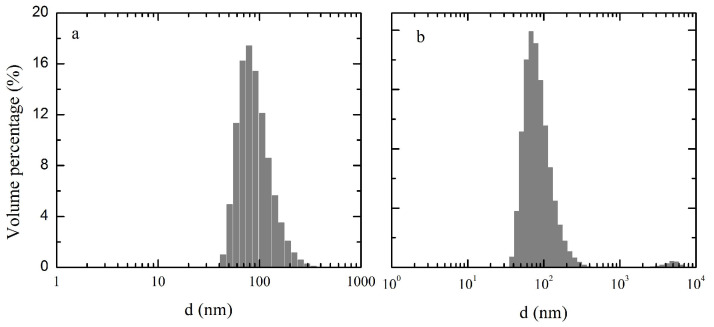
Volume distribution percentage of the NZNPs (**a**) and FZNPs (**b**).

The size distribution of nanoparticles was directly analyzed using DLS to compare NZNPs and FZNPs. The size volume distribution and zeta potential are presented in Figure 3 and Table 1. The particle size distribution histogram is shown in Figure 4. The PDI values for NZNPs and FZNPs were 0.145 and 0.22, respectively, confirming the reliability of the measurements. Significant differences were observed in the particle size distribution of the NZNPs and FZNPs. For small particles (d < 200 nm), the volume percentages were 98.00% and 96.49% for NZNPs and FZNPs. Medium-sized particles (200 nm ≤ d ≤ 1000 nm) accounted for 2.00% of NZNPs and 2.17% of FZNPs. Notably, no large particles (d > 1000 nm) were detected in NZNPs, whereas FZNPs exhibited a 1.43% volume percentage of large particles. Nanoscale particles were successfully obtained from native zein, as confirmed by Zhong and Jin [6]. These results highlight the distinct size distribution profiles between the two nanoparticle types.

The zeta potentials of NZNPs and FZNPs were 37.63 mV and 34.67 mV, respectively, as shown in Table 1. The acidic modification significantly altered the surface charge of flexible zein nanoparticles, as evidenced by zeta potential measurements. This change in surface charge is crucial for optimizing the stability and functionality of nanoparticles, particularly in applications such as bioactive compound delivery and food packaging [32,33]. A more negative or positive surface charge can enhance interactions with target molecules or improve colloidal stability in different environments [34]. However, the addition of acid did not increase the surface charge of the zein nanoparticles, which may be attributed to the increased abundance of carboxyl groups after the acidic deamidation reaction [16,35].

### 3.4. Sensitivity of the FZNPs to a Complex Environment

**Figure 5:**
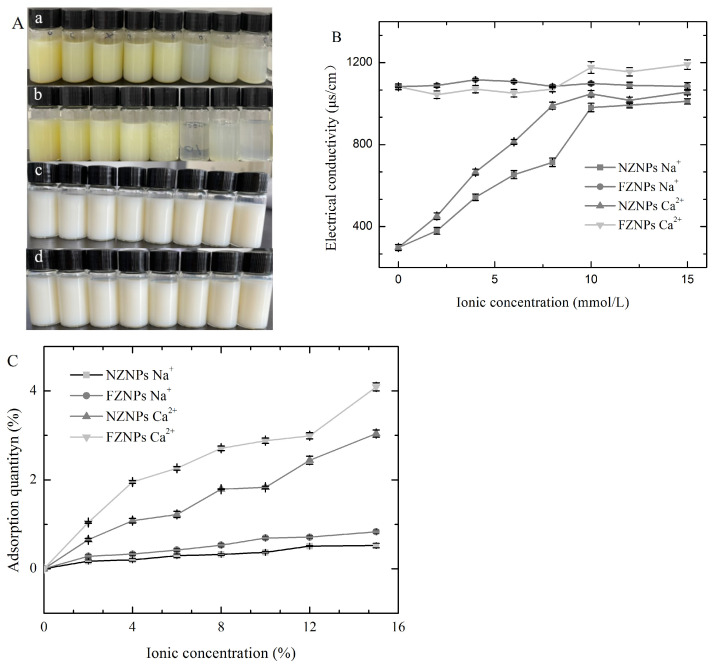
Sensitivity of FZNPs to the complex environment. (**A**) Visual plot of the control and FZNPs in the presence of NaCl and CaCl_2_ at various concentrations. The concentrations of NaCl and CaCl_2_ are 0, 2, 4, 6, 8, 10, 12, and 15 mmol/L from left to right. (**a**) addition of NaCl to the NZNPs, (**b**) addition of CaCl_2_ to the NZNPs, (**c**) addition of NaCl to the FZNPs, and (**d**) addition of CaCl_2_ to the FZNPs. (**B**) Electrical conductivity of the control and FZNPs at different ionic concentrations. (**C**) Adsorption quantity of the control and FZNPs at different ionic concentrations.

Calcium ions (Ca^2+^) and sodium ions (Na^+^) are essential for various biological functions and are widely present in both food and animal tissues. To explore the adaptability of FZNPs, solutions of NaCl and CaCl_2_ at different concentrations (0, 2, 4, 6, 8, 10, 12, 15 mmol/L) were prepared. Both NZNPs and FZNPs were added to these solutions and their behavior was assessed through visual observations and electrical conductivity measurements (Figure 5). The NZNPs solutions appeared yellow due to the presence of xanthophyll (Figure 5A(a,b)), which are strongly associated with hydrophobic proteins and coextracted with zein [3]. In contrast, FZNPs solutions were milky white (Figure 5A(c,d)) because the acidic and thermal treatment used to modify zein destroyed the xanthophylls. As the concentrations of NaCl and CaCl_2_ increased from 0 to 8 mmol/L, the NZNPs solutions remained yellow and transparent (Figure 5A(a,b)). However, when the concentrations of NaCl and CaCl_2_ were further increased from 10 to 15 mmol/L, the solutions became more transparent due to partial precipitation of zein nanoparticles (Figure 5A(c,d)). This indicated that NZNPs did not dissolve well at those ion concentrations and became less stable as suspensions with increasing ionic strength. The transparency of NZNPs increased at higher concentrations of sodium chloride and calcium chloride, with NZNPs showing greater transparency in 10–15 mmol/L CaCl_2_ than in 10–15 mmol/L NaCl. This suggested that CaCl_2_ destabilized NZNPs more than NaCl at high concentrations. In contrast, FZNPs solutions remained milky white across the entire range of 0–15 mmol/L CaCl_2_ and NaCl, indicating different sensitivities of NZNPs and FZNPs to calcium and sodium ions.

Electrical conductivity is a valuable tool for elucidating microstructural changes in self-assembled colloidal systems. To evaluate the adaptability of NZNPs and FZNPs in the presence of monovalent (Na^+^) and divalent (Ca^2+^) ions, their electrical conductivity was measured. The electrical conductivity of NZNPs solutions increased with increasing concentrations of NaCl and CaCl_2_ (from 0 to 15 mmol/L) (Figure 5B). Specifically, the electrical conductivity of NZNPs solutions with CaCl_2_ was higher than that with NaCl. The electrical conductivity of NZNPs solutions increased from 297.87 μs/cm to 1011 μs/cm and from 297.87 μs/cm to 1055 μs/cm as the CaCl_2_ and NaCl concentration increased from 0 to 15 mmol/L, respectively. This increase may be attributed to the shielding of electrical conductivity by calcium ions [36]. In contrast, the electrical conductivity of FZNPs solutions remained relatively high across all concentrations of CaCl_2_ and NaCl. These results suggested that the improved electrical conductivity of FZNPs is likely due to their high adaptability to changing ion concentrations, which is facilitated by the acidic modification that alters the zein structure and increases the abundance of glutamic (Glu) and aspartic acid (Asp) residues [27].

The high adaptability of FZNPs was further demonstrated by their adsorption capacity. Figure 5C shows the adsorption of NaCl and CaCl_2_ by NZNPs and FZNPs at different ionic concentrations. The adsorption quantities of both ions increased with concentration for both types of nanoparticles. However, FZNPs exhibited higher capacities than NZNPs. Specifically, the adsorption of NaCl and CaCl_2_ by NZNPs increased from 0 to 0.52% and from 0 to 3.04%, respectively. While FZNPs adsorbed NaCl and CaCl_2_ at higher levels, increased from 0 to 0.83% and from 0 to 4.09%, respectively. These differences may be attributed to distinct morphologies and particle sizes of NZNPs and FZNPs. The carboxylate groups of Glu and Asp residues have a high affinity for calcium ions [37]. The acid-driven deamidation process used to modify zein introduces more Glu and Asp residues, which can neutralize the negative charges on protein and enhance their capacity to bind calcium and sodium ions [37].

### 3.5. XPS Analysis

**Figure 6:**
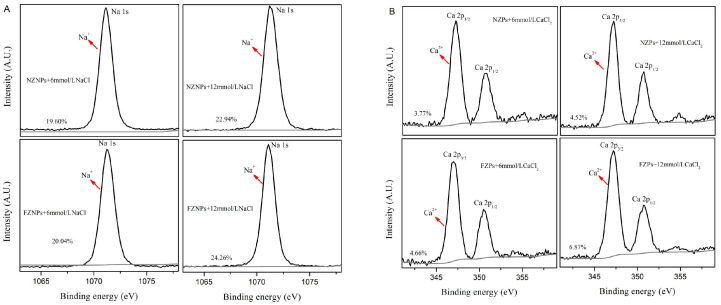
X-ray photoelectron spectrum survey spectra of the FZNPs and NZNPs in presence of 6 and 12 mmol/L NaCl (**A**) and CaCl_2_ (**B**). A.U. = arbitrary units.

To further investigate the binding affinity of FZNPs and NZNPs to Ca^2+^ or Na^+^, we conducted quantitative comparisons using X-ray photoelectron spectroscopy (XPS). The binding quantities were determined by analyzing the peak area obtained from XPS, which reflect the elemental content on the sample surface [23]. The percentages shown in Figure 6 were calculated by normalizing the peak area of each sample to the total peak area of FZNPs and NZNPs, respectively. As the original NZPs and FZPs samples do not contain Na and Ca ions, we did not perform XPS survey spectra analysis on these untreated samples. The results revealed that NZNPs exhibited lower binding capacities for both Na+ (Figure 6A) and Ca^2+^ (Figure 6B) compared to FZNPs at concentrations of 6 mmol/L and 12 mmol/L. Additionally, both FZNPs and NZNPs had higher binding amounts of Na^+^ than Ca^2+^. These differences may be attributed to the interaction of Ca^2+^ with the free carboxyl groups of Asp and Glu residues, forming salt bridges that induce conformational changes in the secondary structure of FZNPs. These changes can alter the distribution of α-helices and β-sheets, exposing more active sites and enhancing the affinity for calcium and sodium ions. Recent studies have demonstrated that surface modification of zein nanoparticles can significantly improve their performance in delivering bioactive compounds, herbal remedies and other applications [32,33,38]. FZNPs, with their higher number of free carboxyl groups, may form more stable chemical bonds with calcium ions, thereby increasing their overall binding capacity. The ability to modulate the surface charge of zein nanoparticles through acidic modification opens new possibilities for designing functional materials tailored to specific applications, such as targeted drug delivery or nutrient encapsulation.

### 3.6. XRD Analysis

**Figure 7:**
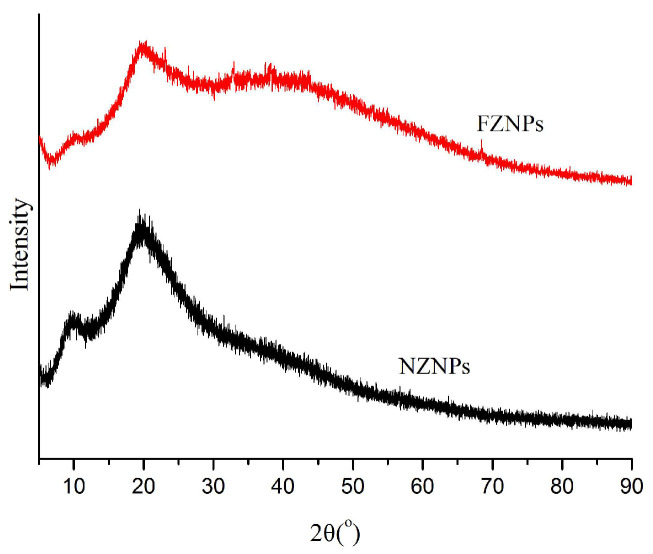
XRD spectra of NZNPs and FZNPs.

The crystal state and diffraction characteristics of NZNPs and FZNPs were examined using XRD patterns. As shown in Figure 7, the diffraction angles (2θ) of NZNPs are 9.6° and 19.3°, respectively. Wei et al. [35] also reported that the individual zein had relatively flat peaks, indicating the amorphous nature of zein. These peaks are relatively broad and flat, lacking sharp characteristic peaks, which is indicative of an amorphous structure. The finding is consistent with the results of Hu et al. [24]. In contrast, the diffraction angles of FZNPs in this study are slightly shifted to 9.9° and 19.7°, indicating that the structure has been altered.

### 3.7. CD Analysis

**Figure 8:**
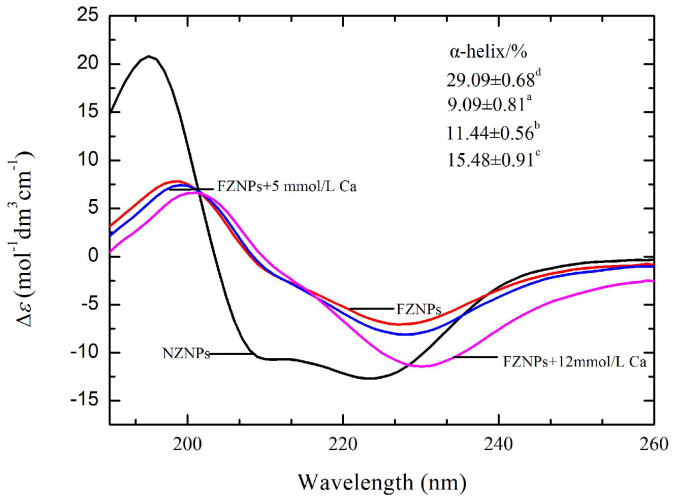
Stacked far−UV CD spectra of NZNPs and FZNPs. The inserted image represents the α−helix content, expressed as means ± standard deviation (SD) of triplicate analyses. Different superscript letters within the same column denote statistically significant differences (*p* < 0.05).

CD spectra were employed to investigate the secondary structure of NZNPs and FZNPs, as shown in Figure 8. Both NZNPs and FZNPs were dispersed in deionized water for comparison. The secondary structure differences were qualitatively assessed by comparing spectra of these zein particles, which have similar dimension and scattering cross-section but differ in absorption properties that significantly affect the signal. The CD spectra of NZNPs exhibited three strong ellipticity values at approximately 195 nm (positive peak), 210 and 222 nm (negative peak), indicative of an α-helix-rich secondary structure [1,16]. In contrast, the FZNPs spectrum showed only two strong ellipticity values at 200 (positive peak) and 228 nm (negative peak), characteristic of a β-sheet-rich secondary structure. To quantitatively assess the α-helix content, Yang’s formula [24] was applied, with the results presented in the insert tables in Figure 8. The α-helix content in the control zein was 29.09%, which is lower than the previously reported value of over 50% for native zein [39]. This reduction may be attributed to the ethanol evaporation process, which has been shown to decrease α-helix content and increase β-sheet content [40,41]. The α-helix content in FZNPs further decreased to 11.44%, likely due to the conversion of α-helix structure to β-sheet, as evidenced by strong ellipticity at 228 nm. The results indicated that the acidic modification significantly altered the secondary structure of zein [16,35]. Additionally, the α-helix content in FZNPs decreased as the Ca^2+^ concentration increased from 0 to 12 mmol/L. These structural changes may contribute to enhanced flexibility of FZNPs.

### 3.8. Amino Acid Compositions

The structural adaptability of FZNPs in complex environment can be attributed to changes in their amino acid composition. The contents of various amino acids in NZNPs and FZNPs are summarized in Table 2. Acidic amino acids, including aspartic acid (Asp) and glutamic acid (Glu), were present in FZNPs at 7.18%, while no acidic amino acids were determined in NZNPs. This difference arises because the amino acid residues in zein, such as asparagine and glutamine, are susceptible to deamidation, a reaction that hydrolyzes these residues to form carboxyl groups and release ammonia [16,27]. In contrast, the basic amino acids (histidine, arginine and lysine) showed only minor differences between NZNPs and FZNPs, with contents of 15.64% and 15.89%, respectively. The composition and sequence of amino acids influence protein structural flexibility [28]. Additionally, the hydrophobic amino acids content differed significantly between control and flexible zein, with values of 38.76% and 36.61%, respectively. Hydrophobic amino acids are known to affect protein flexibility. These changes in the amino acids composition, particularly the introduction of acidic residues and alterations in hydrophobic amino acids, contributed to the secondary structure change in FZNPs. These structural modifications enhance the overall flexibility and adaptability of FZNPs in complex environments. These results are consistent with those reported by Dong et al. [8].

## 4. Conclusions

In conclusion, flexible zein was successfully obtained through acidic modification in 80% ethanol, exhibiting a flexibility degree of 4.89%. The flexibility was further evidenced by the improved emulsion stability of the acid-driven deamidated zein. The FZNPs were prepared using the antisolvent precipitation method. FZNPs displayed a less regular distribution compared to NZNPs, with some FZNPs even appearing fragmented. The particle size distributions of FZNPs differed significantly from that of NZNPs. Additionally, FZNPs demonstrated higher electrical conductivity and enhanced adsorption capacity for ions (CaCl_2_ and NaCl) compared to NZNPs. These findings indicate that FZNPs possess excellent sensitivity to complex environments, likely due to the high ability of secondary structure changes as the environment changes. This conformational adaptability can be attributed to the altered amino acid profile of the flexible zein, highlighting its high structural adaptability. This study provides a promising strategy for designing novel nanoparticles, thereby expanding the potential applications of zein in various fields, such as the delivery of bioactive compounds and herbal remedies systems.

## Figures and Tables

**Table 1:** Particle size volume percentage (%) of the control and FZNPs.

Samples d Range	D < 200nm	200–1000 nm	>1000 nm	PDI	Zeta Potential (mV)
NZNPs	98.00 ± 0.03 ^a^	2.00 ± 0.05 ^a^	0	0.145 ± 0.02 ^a^	37.63 ± 0.17 ^a^
FZNPs	96.49 ± 0.06 ^b^	2.17 ± 0.02 ^b^	1.34 ± 0.07 ^a^	0.220 ± 0.01 ^b^	34.67 ± 0.66 ^b^

Note: Data are presented as means ± standard deviation (SD) of triplicate measurement. Different superscript letters within the same column denote statistically significant differences (*p* < 0.05).

**Table 2:** Amino acid composition of NZNPs and FZNPs.

Samples	Amino Acid Composition (%)		
	Acidic *	Basic **	Hydrophobic ***
NZNPs	0	15.64 ± 1.24 ^a^	38.76 ± 0.46 ^a^
FZNPs	7.18 ± 0.70 ^a^	15.89 ± 0.85 ^a^	36.61 ± 0.56 ^b^

Values are expressed as mean ± standard deviation (SD) of triplicate measurements. Data within the same column marked with different superscript are significantly different (*p* < 0.05, ANOVA). * Acidic amino acids: Asp + Glu; ** basic amino acids: His + Arg + Lys; *** Hydrophobic amino acids: Ala + Pro + Val + Met + Ile + Leu + Phe + Trp.

## Data Availability

The original contributions presented in the study are included in the article, further inquiries can be directed to the corresponding author.
